# Effectiveness of a Peer Support Programme versus Usual Care in Disease Management of Diabetes Mellitus Type 2 regarding Improvement of Metabolic Control: A Cluster-Randomised Controlled Trial

**DOI:** 10.1155/2016/3248547

**Published:** 2015-12-20

**Authors:** Tim Johansson, Sophie Keller, Henrike Winkler, Thomas Ostermann, Raimund Weitgasser, Andreas C. Sönnichsen

**Affiliations:** ^1^Institute of General Practice, Family Medicine, and Preventive Medicine, Paracelsus Medical University, 5020 Salzburg, Austria; ^2^Paris Lodron University, 5020 Salzburg, Austria; ^3^Centre for Integrative Medicine, University of Witten/Herdecke, 58448 Witten, Germany; ^4^Department of Internal Medicine, Wehrle-Diakonissen Hospital, 5026 Salzburg, Austria; ^5^Paracelsus Medical University, 5020 Salzburg, Austria; ^6^Institute of General Practice and Family Medicine, University of Witten/Herdecke, 58448 Witten, Germany

## Abstract

*Aim*. Testing the effectiveness of peer support additionally to a disease management programme (DMP) for type 2 diabetes patients. *Methods*. Unblinded cluster-randomised controlled trial (RCT) involving 49 general practices, province of Salzburg, Austria. All patients enrolled in the DMP were eligible, *n* = 337 participated (intervention: 148 in 19 clusters; control: 189 in 20 clusters). The peer support intervention ran over 24 months and consisted of peer supporter recruitment and training, and group meetings weekly for physical exercise and monthly for discussion of diabetes related topics. *Results*. At two-year follow-up, adjusted analysis revealed a nonsignificant difference in HbA_1c_ change of 0.14% (21.97 mmol/mol) in favour of the intervention (95% CI −0.08 to 0.36%, *p* = 0.22). Baseline values were 7.02 ± 1.25% in the intervention and 7.08 ± 1.25 in the control group. None of the secondary outcome measures showed significant differences except for improved quality of life (EQ-5D-VAS) in controls (4.3 points on a scale of 100; 95% CI 0.08 to 8.53, *p* = 0.046) compared to the intervention group. *Conclusion*. Our peer support intervention as an additional DMP component showed no significant effect on HbA_1c_ and secondary outcome measures. Further RTCs with a longer follow-up are needed to reveal whether peer support will have clinically relevant effects. *Trial Registration*. This trial has been registered with Current Controlled Trials Ltd. (ISRCTN10291077).

## 1. Introduction

The prevalence of type 2 diabetes is estimated to be 6% (42.08 mmol/mol) for the adult population of Austria [1], thus posing a relevant threat to population health. Guideline adherent, structured treatment and management of the disease as proposed by disease management programmes (DMPs) are seen as the best strategy in the prevention of diabetic complications, but evidence from RCTs and systematic reviews on DMPs reveal only modest effects on patient care [2, 3], especially regarding the frequency of clinically relevant endpoints [4]. The Austrian DMP “Therapie Aktiv,” implemented by statutory health insurance in 2007, had no significant effects on metabolic control in a cluster-randomised trial [5] and an open follow-up study [6].

While DMPs have been shown to improve process quality of care [5], they insufficiently address lifestyle changes like physical activity. A logical next step in the improvement of DMPs is therefore the design and implementation of additional components addressing these deficits. Most promising components are interventions to increase physical activity, to decrease caloric intake, and to improve patient education. A Cochrane review on exercise in patients with type 2 diabetes showed that physical activity significantly reduced HbA_1c_ by 0.62% (−16.72 mmol/mol) [7]. Reduced caloric intake and increased physical activity led to weight loss and to a significant improvement of metabolic control and risk factor profile in the Look-AHEAD trial [8, 9]. Patient education has been shown to improve metabolic control in several systematic reviews [10–12]. On the other hand, in all the studies cited above, success and improvement have been moderate and were achieved by quite intensive interventions involving professional support which can hardly be implemented on a population-wide level due to economic and structural reasons.

An alternative might be to emphasize self-management and peer-to-peer motivation instead of professionally dominated educational interventions. Thus a combination of traditional disease management with ongoing peer support may be a promising approach in diabetes care that deserves further evaluation. The idea of peer support goes back as far as the late 1980s, when the impact of diabetes education and peer support were first evaluated [13]. A systematic review of controlled intervention studies on the effect of social and peer support in diabetes identified six randomised trials that all showed some beneficial effects [14] but further studies questioned these early positive results. Although some evidence for the effectiveness of ongoing peer support on metabolic control has been presented [15, 16], a systematic review found this evidence to be too limited to support firm recommendations and calls for further well-designed studies [17]. No such studies have been done on the implementation of long-term peer support programmes added to a traditional DMP. We therefore designed the peer support programme “Aktivtreff Diabetes” as an additional component of the Austrian DMP “Therapie Aktiv” for type 2 diabetes and evaluated the effectiveness of the programme in a cluster-randomised controlled trial.

## 2. Methods

### 2.1. Design

We designed our evaluation study as a pragmatic cluster-randomised controlled trial set in general practices in the province of Salzburg in Austria.

### 2.2. Participants

We invited all 77 surgeries actively administering the DMP “Therapie Aktiv” in Salzburg to recruit participants for the study. All 1327 patients enrolled in the DMP were eligible to participate. We encouraged general practitioners to continuously recruit patients within a recruitment period of eight months (September 2010–April 2011). In addition, Salzburg public health insurance sent an invitation letter to all patients enrolled in the DMP. All patients willing to participate were included in the study after obtaining written informed consent according to the declaration of Helsinki. Exclusion criteria were the following: type 1 diabetes, dementia or major psychiatric illness, advanced neoplastic disease, or other diseases with drastically reduced life expectancy by physician judgement.

### 2.3. Intervention

The intervention was carried out over a period of two years from May 2011 to May 2013 and consisted of four elements which were implemented in addition to the ongoing DMP.

#### 2.3.1. Recruitment of Peer Supporters

General practitioners suggested two peer supporters per intervention group who were made familiar with the details of the study and invited to the peer supporter training. Peer supporters are nonprofessionals who have type 2 diabetes. The peer supporters' main tasks were the following: organization of group meetings and exercise units as well as log of attendance, facilitation of group discussions on diabetes related topics, support of physically weak group members, and motivation of unmotivated participants. Throughout all responsibilities, peer supporters were encouraged to rather give support than advice. They played a crucial role as contact persons for the participants and for the research team. Peer supporters were reimbursed with 10€ per group meeting.

#### 2.3.2. Peer Supporter Training

During the first year of intervention peer supporters received six sessions of training (four hours each). We compiled a standardised curriculum for the training in order to assure reproducibility within the trial and for any application afterwards. Professionals trained peer supporters addressing the following topics: the concept of peer support, organisation of group meetings, physical activity, recommendations for the treatment and management of type 2 diabetes, motivation, medical aspects of diabetes, nutrition, experience, and feedback.

#### 2.3.3. Physical Exercise Meetings

Peer groups consisting of 8–12 participants met once every week for at least one hour of physical outdoor activity such as (Nordic) walking combined with other exercises. The first meetings were facilitated by a physical education trainer to get the groups started and familiarise them with the intervention and exercises. Thereafter, groups met largely autonomously and trainers supported the groups only when needed. The peer groups were regularly provided with instruction sheets (nine in total) showing and explaining exercises for mobilisation, coordination, and strength training.

#### 2.3.4. Peer Group Meetings (Table 1)

[Table tab1]Once a month groups held conversational and educational meetings focusing on personal, social, and emotional topics in the context of diabetes. The meetings were moderated alternately by peer supporters and health professionals and offered the opportunity to ask particular questions and expand and consolidate knowledge about diabetes. To assure standardisation and coverage of the most important topics, we developed a curriculum. It guided participants through subject areas that changed every other month and provided matched topics for every single session. Prior to each meeting, all participants received a newsletter addressing the corresponding topic including the latest scientific findings.

Patients in the control groups received standard care according to the DMP “Therapie Aktiv” which enforces care according to international guidelines regarding monitoring, diagnostics, and treatment of type 2 diabetes.

### 2.4. Outcomes

The primary outcome measure was the difference in change of HbA_1c_ (%, resp., mmol/mol, determined by HPLC using a Menarini HA-8180 HPLC Analyser) from baseline to 24 months between the intervention and control groups. Prespecified secondary outcome measures comprised quality of life EQ-5D-3L index and EQ-5D visual analogue scale (VAS) [18], improved control of cardiovascular risk factors (systolic and diastolic blood pressure measured in the general practices according to standard WHO criteria using locally available gauged blood pressure monitors, total cholesterol, HDL cholesterol, and triglycerides, determined at local laboratories with automated clinical chemistry analysers, and LDL cholesterol, determined using the Friedewald equation), lowering of global cardiovascular risk (UKPDS-Risk Engine 2.0) [19], change in body weight (body mass index (BMI)), and smoking cessation (self-reported).

All primary and secondary outcomes were measured between October 2010 and April 2011 for baseline and between October 2012 and April 2013 for follow-up.

Baseline and follow-up data were recorded pseudonymised by the general practitioners using the structured documentation sheet of the disease management programme “Therapie Aktiv” and case report forms which were then sent to the study centre. We checked all forms for completeness and plausibility. In case of missing or implausible data, we contacted the responsible general practitioner.

### 2.5. Sample Size

We calculated sample size for *α* = 0.05 and *β* = 0.20, proposing 0.5% (−18.03 mmol/mol) difference between intervention and control groups in change of HbA_1c_ from baseline to final examination at 24 months. Using an estimated standard deviation of 1.2% (−10.38 mmol/mol) for HbA_1c_ change, a sample size of 181 patients (91 per arm) was required. Assuming an intracluster correlation coefficient of 0.05 (derived from our previous study [5]) and an average cluster size of 12 patients per peer group, we estimated a design effect of *D* = 1 + (12 − 1) × 0.05 = 1.55. Thus, the sample size increased to 280 patients (140 per arm). Allowing for up to 20% loss to follow-up, the sample size was adjusted to 175 patients per arm or a total of 350 patients.

### 2.6. Randomisation

To assure concealment of allocation, all patients were cluster-randomised by electronic sequence generation using Research Randomizer [20] after completion of recruitment and allocation of patients to prospective peer groups as clusters. Clustering was performed by the study management grouping 8–12 patients living close to each other into a cluster to facilitate face-to-face meetings. If there were a sufficient number of patients in a region, we aimed to group younger patients (<65 years) and older patients (≥65 years) in separate clusters. This resulted in three categories of clusters: category 1: mostly patients <65 years; category 2: mostly patients ≥65 years; category 3: clusters with patients of all ages. Randomisation was stratified by cluster category. When participants signed up and were clustered, neither the study management nor physicians nor patients knew which group would participate in the peer support programme or serve as control, to assure concealment of allocation. Randomisation at the patient level would not have been feasible because the intervention addresses the group.

### 2.7. Blinding

Due to the nature of the intervention, blinding was not possible.

### 2.8. Statistical Analysis

All statistical analyses were performed with IBM SPSS Statistics 20.0. We evaluated our primary endpoint in an intention-to-treat analysis according to the CONSORT guidelines for the reporting of cluster-randomised controlled trials [21]. For missing data regarding HbA_1c_ we applied the method of last available data carried forward. For unadjusted, univariate analysis, we used an independent *t*-test (two-tailed) to detect significant differences between the intervention and the control group. Per-protocol analysis was performed for all secondary outcomes, using independent *t*-tests to detect differences between groups.

In addition to univariate analysis we used a mixed model approach to account for nesting of patients in peer groups and to adjust for covariates. We calculated intracluster correlation coefficients (ICC) and then adjusted for cluster effects, age, and baseline value.

### 2.9. Ethics Approval and Trial Registration

The study protocol was presented to the ethics committee of the province of Salzburg and received unconditional ethics approval on February 24, 2010. The study was registered with current controlled trials on November 17, 2010 (ISRCTN10291077).

## 3. Results

### 3.1. Participants, Recruitment

**Figure 1:**
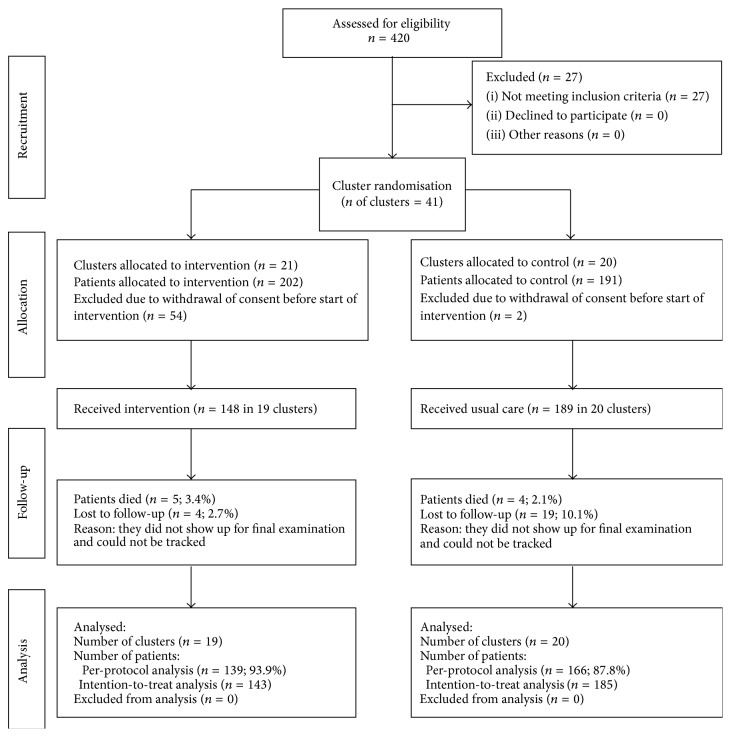
CONSORT diagram for recruitment and follow-up of clusters and participants.

Forty-nine (63.6%) of the eligible general practices (*n* = 77) recruited patients for the study. A total of *n* = 420 (29.6%) of all eligible patients (*n* = 1327) initially signed up. Twenty-seven patients did not meet inclusion criteria. Fifty-six patients withdrew consent after randomisation (54 interventions, 2 controls), and 9 patients died (5 interventions, 4 controls), leaving 328 patients for intention-to-treat analysis (intervention group: *n* = 143; control group: *n* = 185).  Figure 1 shows the CONSORT flow diagram of the study. For 23 patients (intervention group: *n* = 4; control group: *n* = 19) final HbA_1c_ had to be imputed using the last available data carried forward method due to loss to follow-up.

Recruitment took place from September 2010 to April 2011 and the intervention ran from May 2011 until May 2013. All practices and peer supporters continued to participate during the whole study period.

### 3.2. Process Evaluation

Peer supporters visited median 5 (0–6) of the six peer supporter training sessions. The median number of physical activity meetings per group was 86 (1–104) with an achievable maximum of 104 times (once per week for two years). The median number of physical activity meetings of individual patients was 23 (0–90). Physical education trainers supported the groups in 11% of all performed physical activity meetings (148 of 1344).

Peer groups met 12 (0–14) times for all educational and conversational meetings (67% of 15 possible meetings), and of these 8 (0–8) were supported by a professional. Individual patients participated in 4 (0–14) of these meetings (34% of all meetings). In total, 178 group meetings were performed by the peer groups, of which 126 (72.4%) were supported by a professional.

### 3.3. Baseline Data

The baseline characteristics of the participants were balanced between the study groups (Table 2).

### 3.4. Follow-Up Results

Using univariate analysis (independent *t*-test) or mixed models adjusting for baseline values and cluster effects, we found no significant differences between the intervention group and the control group regarding our primary and most secondary outcomes (Tables 3 and 4). Quality of life decreased in the intervention group and slightly improved in the control group, with a significant difference between groups in favour of the controls (EQ-5D index and EQ-5D VAS, Table 3). Only the difference in EQ-5D-VAS remains significant after adjustment for baseline value and cluster effects (Table 4). There was no significant difference between the intervention and control groups regarding smoking cessation. Four of 124 patients (3.2%) in the intervention group stopped smoking compared to two of 146 (1.4%) patients in the control group (*p* = 0.649).

For safety reasons we calculated the relative risk of the intervention for death or cardiovascular events (per-protocol analysis). No significant differences between the two groups could be seen (Table 5).

## 4. Discussion

Our study showed that a group based peer support intervention as an additional component to a traditional disease management programme on type 2 diabetes in general practice is feasible. Although peer support appears to be a very promising approach, our intervention did not significantly improve clinical outcomes or risk profile. A slight negative effect could even be seen regarding health related quality of life.

As demonstrated by the UKPDS [22], HbA_1c_ levels gradually worsen over time with a rise of about 0.1% (−22.4 mmol/mol) per year. We therefore postulated an increase of HbA_1c_ of about 0.2% (−21.31 mmol/mol) in the control group and a small decrease of HbA_1c_ in the intervention group, basing our sample size calculation on a net difference of 0.5% (−18.03 mmol/mol) in HbA_1c_ change over the two years of follow-up. Our supposition was only partly fulfilled: While HbA_1c_ rose in controls it did not decrease in the intervention group but was only kept unchanged. We therefore missed to show a significant effect of our peer support intervention on our primary endpoint. A larger sample and a longer follow-up would be needed to show whether the peer support intervention can significantly prevent the rise in HbA_1c_ in the long run.

Our findings may at least partially be due to the well-controlled baseline values, leaving little room for improvement. Although general practitioners had been asked to recruit all patients with type 2 diabetes consecutively, we suspect that recruitment was performed differentially giving priority to well-controlled patients. Also, the intensity of the intervention may have been too low to demonstrate an effect on HbA_1c_. Increased professional support would have intensified the intervention, but this would have been contradictory to our intention of implementing group based peer support with a main focus on patient self-management. Due to withdrawal of consent, some groups were smaller than planned with a potentially negative impact on group dynamics.

Although quality of life was a predefined secondary outcome in our study we do not suggest to overestimate the marginally significant negative effect seen in our results. Firstly, a difference of 4 points on a VAS with 100 points does not seem clinically relevant. Secondly, this result could very well be due to chance in multiple testing. On the other hand, a decrease in quality of life as a consequence of our intervention cannot be ruled out and should be carefully considered as a possible detrimental effect of the programme. A reduction in well-being was also described in the study of Smith et al. who evaluated a peer support programme for type 2 diabetes mellitus in Ireland [23]. Smith postulates that peer support could cause peer supporters to focus on negative experiences which may have a negative effect on all participants. The increase of physical activity could also lead to discomfort in patients not used to exercising, thus compromising quality of life.

### 4.1. Comparison with Other Studies

Our results are in line with the majority of peer support trials that could not demonstrate a positive effect on HbA_1c_ [23–27]. Only very few controlled studies could show that peer support has a significant impact on glycaemic control in patients with type 2 diabetes [16, 28, 29]. All of the three positive studies were of short duration (≤6 months) and tested quite specific interventions like counselling via telephone or an online programme. According to the aforementioned systematic review, studies on peer support are in general heterogeneous in terms of setting, intervention, study design, length of follow-up, and outcome measures, and often the quality is low [17]. We could only identify one long-term randomised controlled trial exploring the effect of a peer group based intervention. This Irish study, like our study, showed disappointing results regarding the effect of peer support on metabolic control and, as mentioned above, also detected a possible negative effect on quality of life [23]. Compared to our trial, the intervention examined in Smith's study was of very low intensity: Study participants had only nine meetings with peer supporters in two years, and peer supporters only had two evening training sessions. We hypothesized that our more intensive peer support intervention would have a more notable effect on HbA_1c_. However, our results are consistent with the Irish peer support intervention. It remains unclear how to design and implement peer support as a structured intervention to effectively optimise metabolic control.

Some peer support studies for type 2 diabetes have shown benefits for participants using other outcome measures compared to HbA_1c_ such as healthier eating habits [27, 30, 31], health distress [16, 27, 28], blood pressure [32], BMI [30], or depression [31]. For all of these outcomes there exist other studies which did not find any effect [17], and in their systematic review Dale et al. draw the conclusion that no consistent evidence exists that supports a general benefit of peer support interventions [17].

### 4.2. Strengths and Limitations

This cluster-randomised controlled trial is one of the largest randomised trials on group peer support, and no existing controlled study provided longer follow-up than ours. Our peer support intervention was well designed, and process evaluation assured that the intervention was largely delivered as planned. Loss to follow-up (6.8%) was low, and the amount of missing data was acceptable for a pragmatic trial. Diabetes care was well structured by the existing DMP in both the intervention and the control group, assuring that any differences in outcome could be attributed to the intervention and not to differences in standard health care delivery. All of these characteristics of our study assure a high degree of internal validity.

Nonetheless there are several sources of possible bias possibly compromising our study results. To avoid selection bias and to assure concealment of allocation, neither physicians nor patients knew at the time of signing up whether they would participate in the peer support programme or serve as controls. A number of patients dropped out after randomisation into the intervention group, presumably willing to participate in the study only as controls. Selection bias may have occurred here.

As mentioned above, differential recruitment of healthy patients by the general practitioners may have led to sampling bias or healthy user bias. There was little room for improvement of metabolic control in our study population which thwarted our power calculation. HbA_1c_ improvement by 0.5% is not a clinically realistic expectation when starting from a baseline HbA_1c_ of 7%. Our peer support intervention might therefore be more effective in patients with poor glycemic control. On the other hand, peer support is probably not suitable for all patients, and intentionally recruiting less well patients may lead to low participation rates in the group sessions, thus also compromising a possible effect.

Preselecting participants by restricting inclusion to patients enrolled in the DMP might be another source of sampling bias. In Salzburg, only about 10% of all patients with type 2 diabetes have been enrolled in the DMP at the beginning of our study. Thus external validity of our study is limited due to various reasons even though internal validity is high.

Attendance rates of the group sessions in our study were good but not excellent. Some patients only attended the discussion meetings and avoided the exercise meetings, thus compromising their chance to improve metabolic control by physical activity. Although patients and peer supporters were instructed and motivated to do additional exercising at home, most patients probably only participated in the physical activity group meetings once a week. This intervention may have been not sufficiently intense to exert an influence on our primary endpoint.

As the intervention made it necessary to preform groups of patients living close to each other, and due to the group based intervention, randomisation on the patient level was not possible. Bias due to cluster effects can be minimized by multilevel modelling but there remains a risk of cluster bias due to undetected confounders.

Our power calculation was based on a 0.5% (−18.03 mmol/mol) difference in HbA_1c_ reduction between intervention and control which was not achieved. Nonetheless HbA_1c_ increased by 0.1% (−22.4 mmol/mol) in controls, and this increase was apparently avoided by the peer support intervention. A much larger sample size and longer follow-up would be needed, though, to make this result significant.

As we know from the ACCORD study, low HbA_1c_ levels are not necessarily related to better outcome [33]. Therefore it might be postulated that HbA_1c_ is not a suitable outcome measurement in studies of type 2 diabetes. We agree to this postulate in intervention studies focused on drugs, but we believe that HbA_1c_ still is an acceptable outcome measure in studies focused on lifestyle changes. We evaluated clinically relevant outcomes like event rates and mortality as safety measures in our study, but follow-up certainly was not long enough to expect any significant effects of the intervention here.

### 4.3. Conclusions and Policy Implications

A group based peer support intervention as an additional component of a disease management programme on type 2 diabetes is feasible. It enables general practitioners to offer additional support to patients willing to be active and change their lifestyle, it requires minimal effort from the general practitioners, and it can be offered at low cost as the intervention is mainly carried out by the patients themselves. Our intervention tends to maintain HbA_1c_ while it gradually worsens in controls as has been shown in other studies like the UKPDS. Larger randomised controlled trials with a longer follow-up are needed to demonstrate the significance of this finding and to evaluate the effects of peer support on clinically relevant endpoints. To date, evidence is insufficient to give a general recommendation regarding the implementation of peer support programmes, but we believe that the concept provides an additional opportunity for chronically ill patients and therefore deserves further research.

## Figures and Tables

**Table 1 tab1:** Topics for the group meetings and newsletters.

Year	Month	Topic/newsletter	Professional
2011	10–12	Healthy diet: dietary change step by step	Nutritionist

2012	01	Self-motivation and group motivation	—
	02	Lifestyle changes	Psychologist
	03	Daily self-management, medical checks	—
	04	Diabetes: therapy, blood glucose measurement	General practitioner
	05	Sweeteners	—
	06	Weight loss, weight control	Nutritionist
	07	Physical activity in daily routine	—
	08	Physical activity and motivational problems	Sports scientist
	09	Cardiovascular risk management	General practitioner
	10	Prevention of diabetic complications	—
	11	Glycaemic index and glycaemic load	Nutritionist
	12	Prevention of weight gain at Christmas	—

2013	01	Diabetes and depression	Psychologist
	02	Diabetes and alcohol; smoking cessation	—

**Table 2:** Baseline characteristics of participants.

	*n* (I/C)^a^	Intervention *n* = 148	Control *n* = 189
Age (years, SD)	148/189	62.2 (8.8)	63.6 (10.8)

Female (*n*, %)	148/189	76 (51.4)	97 (51.3)

Duration of diabetes (years, SD)	145/178	8.4 (7.1)	7.0 (5.6)

Smokers (*n*, %)	141/183	17 (12.1)	22 (12.0)

Married (*n*, %)	145/182	99 (68.3)	121 (66.5)

Low level of education^b^ (*n*, %)	143/181	126 (88.1)	164 (90.6)

Retired (*n*, %)	145/183	95 (65.6)	126 (68.8)

Living alone (*n*, %)	144/178	30 (20.8)	37 (20.8)

^a^Variation of *n* due to missing values; I = intervention group, C = control group.

^b^Only grade school, apprenticeship.

**Table 3:** Clinical outcomes at baseline and follow-up by study group.

	Intervention			Control			Mean difference between groups^b^ (95% CI)	*p*-value^c^
	*N* ^a^	Mean (SD) baseline	Mean (SD) follow-up	*N* ^a^	Mean (SD) baseline	Mean (SD) follow-up		
Primary endpoint								

HbA_1c_ (%)	143	7.02 (1.25)	7.05 (1.10)	185	7.08 (1.25)	7.21 (1.31)	0.10 (−0.14 to 0.35)	0.41

Secondary endpoints								

*Laboratory results (mg/dL)*								
Creatinine	139	0.86 (0.23)	0.90 (0.25)	162	0.96 (0.33)	1.00 (0.64)	0.01 (−0.07 to 0.10)	0.77
Triglycerides	139	150.8 (86.4)	147.9 (81.1)	164	151.7 (94.3)	147.2 (83.7)	−1.5 (−20.2 to 17.2)	0.88
Cholesterol	139	189.5 (40.1)	187.1 (40.3)	164	190.5 (44.8)	184.4 (40.6)	−3.6 (−12.8 to 5.5)	0.43
HDL	139	54.9 (14.4)	57.1 (18.7)	163	54.8 (16.4)	55.0 (15.0)	−2.0 (−4.9 to 0.9)	0.17
LDL	136	106.3 (35.9)	100.3 (37.0)	161	106.7 (38.9)	99.1 (35.7)	−1.1 (−9.3 to 7.1)	0.79

*Anthropometric measurements*								
BMI (kg/m^2^)	133	31.0 (5.3)	30.7 (5.3)	159	30.3 (4.8)	29.9 (4.9)	−0.1 (−0.6 to 0.3)	0.65
Systolic blood pressure (mmHg)	128	136.0 (15.7)	136.0 (15.7)	154	137.2 (17.9)	136.3 (15.8)	−1.0 (−5.2 to 3.2)	0.65
Diastolic blood pressure (mmHg)	128	80.8 (9.1)	80.4 (8.5)	154	80.4 (10.0)	80.8 (8.6)	0.8 (−1.7 to 3.2)	0.52

*UKPDS-Risk Engine: 10-year risk* ^f^								
CHD^d^	76	15.0 (9.3)	16.9 (11.0)	85	15.3 (9.9)	17.1 (9.6)	0.0 (−1.9 to 1.9)	0.99
Fatal CHD	76	10.2 (7.5)	12.2 (9.3)	85	10.2 (8.6)	12.1 (8.4)	−0.1 (−1.6 to 1.3)	0.85
Stroke	76	8.9 (7.3)	11.3 (9.4)	85	9.1 (8.4)	11.2 (9.2)	−0.4 (−1.2 to 0.3)	0.27
Fatal stroke	76	1.3 (1.3)	1.8 (1.8)	85	1.4 (1.5)	1.6 (1.4)	−0.2 (−0.5 to −0.0)	0.047

*Quality of life (EQ-5D)*								
Index	128	0.90 (0.16)	0.87 (0.20)	149	0.88 (0.19)	0.88 (0.19)	0.04 (−0.0 to 0.1)	0.051
VAS^e^	117	75.1 (17.0)	72.8 (20.0)	130	70.9 (17.4)	73.7 (18.8)	5.2 (0.6 to 9.8)	0.03

^a^Variation of *n* due to missing values; BL = baseline; FU = follow-up.

^b^Mean difference between groups is calculated by subtracting mean pre-post-difference of the control group from mean pre-post-difference of the intervention group.

^d^Independent *t*-test, unadjusted.

^4^CHD = coronary heart disease.

^e^VAS = visual analogue scale.

^f^The reduced *n* is due to the fact that the UKPDS-risk engine can only be applied to patients in primary prevention.

**Table 4:** Differences between groups regarding primary and secondary outcome measures, adjusted for baseline values, age, and ICC.

	*n* (I/C)	Mean difference between groups^b^	ICC^a^	*p* value
		(95%-CI)		
Primary endpoint				

HbA_1c_ (%)	143/185	0.14 (−0.08 to 0.36)	−0.05	0.22

Secondary endpoints				

*Laboratory results (mg/dL)*				
Creatinine (mg/dL)	139/162	0.00 (−0.09 to 0.08)	−0.01	0.92
Triglycerides (mg/dL)	139/164	0.17 (−15.54 to 15.88)	0.00	0.98
Total cholesterol (mg/dL)	139/164	−2.28 (−10.05 to 5.48)	0.05	0.56
HDL cholesterol (mg/dL)	139/163	−1.94 (−4.69 to 0.81)	0.17	0.17
LDL cholesterol (mg/dL)	136/161	−0.37 (−7.48 to 6.74)	0.06	0.91

*Anthropometric measurements*				
BMI (kg/m^2^)	133/159	−0.15 (−0.58 to 0.29)	−0.03	0.51
Systolic blood pressure (mmHg)	128/154	−0.56 (−3.96 to 2.84)	−0.05	0.75
Diastolic blood pressure (mmHg)	128/154	0.51 (−1.39 to 2.41)	−0.02	0.60

*UKPDS-Risk Engine: 10-year risk* ^e^				
CHD^c^	76/85	0.10 (−1.75 to 1.95)	0.03	0.92
Fatal CHD	76/85	−0.09 (−1.53 to 1.36)	0.03	0.91
Stroke	76/85	−0.43 (−1.07 to 0.22)	−0.03	0.20
Fatal stroke	76/85	−0.22 (−0.44 to 0.00)	−0.07	0.053

*Quality of life (EQ-5D)*				
Index	128/149	0.04 (−0.00 to 0.08)	−0.01	0.08
VAS^d^	117/130	4.30 (0.08 to 8.53)	0.01	0.046

^a^ICC = intracluster correlation coefficient.

^b^Δ adjusted = adjusted mean difference, calculated using mixed models. Mean difference is calculated by subtracting mean pre-post-difference of the control group from mean pre-post-difference of the intervention group. Positive values indicate higher reductions in the intervention group compared to controls. Negative values indicate an increase in the intervention group compared to controls.

^c^CHD = coronary heart disease.

^d^VAS = visual analogue scale.

^e^The reduced *n* is due to the fact that the UKPDS-risk engine can only be applied to patients in primary prevention.

**Table 5:** Cardiovascular events and mortality.

Event	Intervention *n* = 139	Control *n* = 166	Relative risk	95% CI
Death	5	4	1.49	0.41 to 5.45
Myocardial infarction	2	4	0.60	0.11 to 3.21
Bypass or stenting	8	6	1.59	0.57 to 4.48
Stroke	2	1	2.39	0.22 to 26.06
Any cardiovascular event	9	8	1.34	0.53 to 3.39
Any cardiovascular event or death	13	12	1.29	0.61 to 2.74

## Abbreviations

ACCORD: Action to Control Cardiovascular Risk in Diabetes
BMI: Body mass index
CHD: Coronary heart disease
DMP: Disease management programme
HbA_1c_: Haemoglobin A_1c_
HDL: High density lipoprotein
LDL: Low density lipoprotein
UKPDS: United Kingdom Prospective Diabetes Study
VAS: Visual analogue scale.
